# Challenges and opportunities for the reintegration of sex-trafficking survivors into Nepalese communities

**DOI:** 10.1080/16549716.2025.2522489

**Published:** 2025-07-09

**Authors:** Ranjila Joshi, Pernille Tangaard Andersen, Arja R. Aro, Mette Rømer, Subash Thapa, Leena Eklund Karlsson

**Affiliations:** a Unit for Health Promotion, University of Southern Denmark, Esbjerg, Denmark; b Department of Public Health, University of Southern Denmark, Odense, Denmark; c University of Turku, Turku, Finland; d Health Inequality and Resilience Research Institute, Kathmandu, Nepal; e Department of Sociology and Social Work, Aalborg University, Aalborg, Denmark; f Rural Health Research Institute, Charles Sturt University, Orange, Australia

**Keywords:** Human rights, non-governmental organizations, reintegration, sex trafficking, trauma-informed care, stigma reduction

## Abstract

**Background:**

Although Nepal ratified the Palermo Protocol in 2020, significant challenges persist in fully meeting the protocol’s standards on preventing trafficking, protecting survivors, prosecuting traffickers, and reintegrating survivors. Most importantly, given the complexity surrounding the reintegration of sex trafficking survivors, a comprehensive understanding of the factors influencing successful reintegration is crucial.

**Objective:**

This study explored the challenges and opportunities associated with reintegrating survivors of sex trafficking into Nepali communities.

**Methods:**

This was an exploratory, descriptive qualitative study in which semi-structured interviews were conducted with 15 purposively selected non-governmental organization (NGO) workers and service providers. Thematic analysis was conducted to analyze the data.

**Results:**

Our findings revealed that existing social and contextual barriers included a lack of familial acceptance, social stigma, ineffective legal and support systems, and safety and security risks for both survivors and those providing support. Within the programs facilitating reintegration, factors such as poor service satisfaction, lack of trust, restricted living conditions in the shelters, and inadequate confidentiality practices hindered program effectiveness. Opportunities for successful reintegration included family support and acceptance, employment as social workers/activists, and well-coordinated support systems, which, however, were only accessible to a limited number of survivors.

**Conclusions:**

This study provides insights into how social and program-specific factors, as well as existing opportunities, influence the effective reintegration of survivors. Ensuring adequate funding to scale up current reintegration programs, incorporating strategies to address community stigma, and strengthening institutional capacities could help more women achieve successful reintegration.

## Paper Context

- **Main findings** Survivors of sex trafficking in Nepal face significant reintegration barriers, including social stigma, lack of familial support, ineffective legal systems, and limited program effectiveness, but opportunities such as family acceptance and coordinated support exist for some.
- **Added knowledge** This study highlights the role of social and programmatic factors in the reintegration of trafficking survivors and identifies gaps in service delivery, confidentiality, and safety that hinder successful outcomes.
- **Global health impact for policy and action** Enhancing reintegration programs by addressing stigma, improving institutional support, and increasing funding can inform global strategies to better support trafficking survivors in their return to communities.

## Background

Human trafficking involves the illegal transportation or sale of human beings across or within countries, typically for monetary or other compensation, often using deception, coercion, or violence for purposes such as prostitution, slavery, or bonded labor [1]. The International Labor Organization (ILO) estimates that approximately 5 million people worldwide are survivors of sexual exploitation, with 98% being women and girls [2]. The trafficking of young women and girls for forced sex work and other exploitative conditions is a rapidly growing industry in many countries, including Nepal, India, Thailand, the Philippines, Israel, the United States, and Canada [3]. Sex trafficking is associated with a significant increase in both physical and mental health issues, including a heightened risk of diseases such as HIV and Hepatitis C [4,5].

In Nepal, an estimated 7,000 women are trafficked each year, primarily to brothels in major Indian cities, such as Mumbai and New Delhi, where around 200,000 Nepali women and girls are reportedly engaged in forced labor [4,6–9]. Survivors often escape from Indian brothels to find safety from the severe physical and mental trauma they endure daily [7,10]. However, there are no official data on how many sex-trafficked women return to Nepal, making it difficult for program workers to estimate the number of women in need of reintegration services [9]. To support returnees, Nepal has introduced various measures, including surveillance to identify survivors, rescue operations, and new regulations aimed at protecting trafficking survivors [11–13]. Although Nepal became the 176^th^ country to ratify the Palermo Protocol in 2020, it has yet to meet key standards for preventing trafficking, protecting survivors, prosecuting traffickers, and reintegrating survivors [11].

Reintegration is a critical component of the anti-trafficking response. In Nepal, non-governmental organizations (NGOs) play a crucial role in implementing reintegration programs at the community level. These programs offer rehabilitation homes, education, skill training, medical services, and referrals for survivors. Additionally, community-level initiatives focus on family reunification, societal reintegration campaigns, follow-ups, and financial support through business grants. However, working with trafficking survivors and their families presents significant challenges due to the sensitive nature of the issue and potential shortcomings in program implementation, which may exacerbate their circumstances [14].

Understanding the social context of successful reintegration and gaining evidence-based insights into how reintegration programs function and can be implemented is essential [15]. This is particularly important for preventing the risk of re-trafficking, a well-documented issue among returnees, and for supporting their health and well-being [16]. Therefore, this study explored the challenges and opportunities associated with the effective reintegration of survivors of sex trafficking into Nepalese communities.

## Methods

### Study design

This study opted for an exploratory, descriptive qualitative (EDQ) study design to explore the opportunities and challenges of re-integrating survivors of sex trafficking. The EDQ approach is a widely cited research tradition that allows for a deeper understanding of the topic under investigation and enables study participants to contribute to the development of new knowledge [17]. Ethical approval for this study was obtained from the Ethical Review Committee of Nepal Health Research Council (Reg no.182/2018).

### Participants, sampling, and recruitment

Participants consisted of NGO workers and service providers involved in the reintegration of sex trafficking survivors for at least 2 years. The study focused solely on NGO workers because they play a central role in reintegration efforts, providing direct services, advocacy, and policy engagement. Survivors of sex trafficking, their family members, and government stakeholders were not included due to ethical concerns regarding survivors’ vulnerability and reluctance to disclose their trafficking history.

A list of potential NGOs from Kathmandu, Lalitpur, and Makawanpur was compiled, along with reintegration workers based either in the central office or in the field outside these districts. A snowball sampling technique was used to identify the participants. Kathmandu and Lalitpur were selected due to their concentration of resources, legal offices, and international organizations supporting reintegration programs, while Makawanpur was included for its high trafficking prevalence and active reintegration initiatives, ensuring diverse perspectives while remaining logistically feasible.

Recruitment was carried out through direct contact via phone or email using publicly available sources, professional networks, and referrals. Identified organizations and participants were invited to participate in semi-structured interviews. Informed written consent was obtained from the referred participant and their organization before any contact was initiated, ensuring confidentiality and allowing participants to opt out at any stage. All participants who agreed to be interviewed confirmed having knowledge and/or experience in implementing anti-trafficking interventions and of having contact with women or families affected by trafficking [18].

A total of 20 NGO workers from 13 different NGOs were approached, and 15 of them provided written informed consent to participate in the study. Of these, four participants were from NGOs based in Kathmandu, three were from Lalitpur, and eight were from Makawanpur.

### Data collection

Face-to-face, semi-structured interviews were conducted between June and August 2018. An interview guide was initially developed in English and translated into Nepali by a bilingual translator. To ensure clarity and cultural relevance, the translated version was reviewed by another bilingual researcher. Additionally, the guide was pilot-tested with an NGO worker to refine the wording and improve comprehension. While a formal back-translation was not conducted, feedback from the pilot test was incorporated to enhance accuracy. The final interview guide included questions about participants’ work experiences, their experiences of working with trafficked women, re-integration efforts, ongoing interventions, implementation strategies, the role of different stakeholders, and specific opportunities and challenges related to program delivery.

The principal researcher (RJ), a female Nepalese researcher familiar with the local language and the sensitivity of this issue, conducted all the interviews. To ensure confidentiality and participant comfort, all interviews were conducted in private office spaces within the NGO premises. No financial compensation was provided to participants, and efforts were made to schedule interviews at locations convenient for them minimizing travel costs. One female research assistant took notes during each interview. On average, the interviews lasted 1 hour, and all were tape-recorded.

The 15 participants – 10 females and 5 males – and the majority had work experiences between 10 and 14 years in reintegration, and most had university degrees (Tables 1 and 2).

**Table 1.** Professional roles and sex of interview participants.

Professional Role	Female	Male	Total
Program management	6	4	10
Advisory	1	1	2
Advocacy	3	0	3
Total	10	5	15

**Table 2.** Years of experience of interview participants.

Years of Experience	Number of Participants
1–4 years	2
5–9 years	2
10–14 years	6
15+ years	5

### Data analysis

All audio recordings were transcribed independently into English by two research assistants, and the principal researcher cross-checked both versions for accuracy. We opted for reflexive thematic analysis (Braun and Clarke) to explore participants’ perspectives, identify similarities and contrasts, and develop a detailed understanding of the challenges and opportunities in reintegration. Themes were revisited iteratively to ensure they accurately captured the shared meanings expressed by the participants [19].

At first, a thorough reading of the transcripts and labelling initial codes were conducted, with each code expressing a concept. The process was iterative, with the researcher actively reflecting on how their perspectives influenced the coding. Then, broader conceptual themes (broader codes) emerged by merging two or more initial codes based on recurring ideas or the relationships between them. The broader codes were checked for patterns across all the data to deepen our understanding of these phenomena. This reflective process involved interpreting participants’ beliefs, experiences, and the meanings they attributed to the phenomena. NVivo-10 software was used to support the analysis.

## Results

Our analysis identified seven challenges and three opportunities related to the reintegration of sex trafficking survivors into their communities. Challenges included: ‘ineffective legal and support systems’, ‘safety and security risks’, ‘poor confidentiality’, ‘social stigma and labeling’, ‘poor satisfaction with NGO services’, ‘lack of trust in NGO services’, and ‘restricted living conditions within the NGO shelters’. The opportunities included: ‘‘family support’, ‘returnee women working as social workers/activists’, and ‘coordinated support systems’.

### Challenges of reintegrating sex-trafficking survivors into communities

Challenges included factors or conditions that negatively affected the NGO workers’ motivation, hindered program implementation, and impacted survivors’ overall reintegration process.

#### Ineffective legal and support systems

Most participants characterized the legal system as weak, particularly due to its inability to enforce laws against trafficking and punish traffickers. Those convicted often received short-term sentences of only 2–3 years, despite Nepali law mandating life imprisonment for such crimes in line with the Palermo Protocol, which Nepal ratified in 2020. Many participants explained that the legal system was highly ineffective due to several factors: lack of survivor-witness protection, survivors, and their families being bribed by traffickers, survivors being forced to produce documents for prosecution, lengthy waiting time for court hearing processes, and the inability to punish traffickers due to the involvement of politically influential individuals within trafficking networks. Often, the traffickers had political protection and were bailed out quickly.

For traffickers, there should be provisions for lifetime imprisonment. But in reality, they often receive very short sentences, just 2 to 3 years, and then they are released to commit crimes again. (Participant of program management role)

Firstly, there is a strong network of traffickers and their supporters, who may have political backing. It was also reported in the news that a sex trafficking broker, who was the president of one of the village development committees, was released from prison after paying a certain amount to the police officers. (Participant of program management role)

Additionally, participants reported being treated poorly when requesting social services for returnees at police stations and NGOs. They also noted inadequate attention to healthcare needs in the returnee response programs, with some cases where services were denied altogether.

We picked up a woman from the airport who had wounds all over her body and was severely malnourished. We took her to the hospital, but they refused to provide care, stating that she needed to be reported as a police case first. We tried two other hospitals, but both also rejected her. We cannot simply leave such cases in the hospital, assuming they will receive care without further issues. So, we must take responsibility until the end. (Participant of advisory role)

#### Safety and security risks

Participants highlighted the ongoing safety and security risks faced by both survivors and NGO workers. Traffickers often posed a direct threat, including physical violence, blackmail, and even death threats. NGO workers also described living in fear of retribution, with some survivors being coerced into silence or withdrawing from the reintegration process due to threats from traffickers. Some participants expressed disappointment as the traffickers were often protected by political leaders. They further explained that survivors often faced threats from traffickers who wanted to control or re-exploit them, as well as from members of the community with harmful prejudices. Two participants shared their experiences as below:

The next thing to suffer from is death threats. I will have to live with fears for my whole life. (Participant of program management role)

A survivor approached me, asking for help in filing a case against traffickers. Somehow, the traffickers found out. Someone threatened me over the phone, saying they would break my hands and legs. This made me question whether I should stop helping the returnees and withdraw from the case. I was also deeply worried that they might harm my children and family. (Participant of program management role)

#### Poor confidentiality

In some cases, returnees were required to disclose personal information, including sensitive details about their trafficking experiences, which exposed them to stigma. Three participants explained that NGO shelters would only accept returnees if they disclosed their backgrounds and shared information about their families for registration. However, many survivors feared that the information they shared might be leaked, and as a result, some women withheld critical details or provided inaccurate information. This reluctance to disclose personal history complicated the identification and assistance processes.

When survivors arrive, some do not share accurate information about themselves or their families. Lacking this information makes it very difficult to locate their families. In some cases, knowing the correct details allows us to assist them with administrative tasks, such as obtaining identification cards. At times, I felt frustrated, but then I remind myself that they may need more time to accept their situation and reveal their identity. (Participant of program management role)

#### Social stigma and labeling

Survivors were often labeled as trafficked women, prostitutes, or victims, which led to feelings of shame and isolation. These labels were especially problematic for women from marginalized groups, such as Dalits, who already experienced societal discrimination. Survivors feared that revealing their backgrounds would lead to further stigmatization, with one participant describing how a returnee from a lower caste was forced to hide her identity to avoid further societal rejection.

For Dalit [lower caste] women, they hide their surname because society does not accept a lower caste woman who has lived in India [brothels]. They are perceived differently and live with shame. There is double labeling: one for being from a low caste and another for having worked as a prostitute in India. To cope with this, they often change their original name. (Participant of advocacy role)

#### Poor satisfaction with NGO services

Two participants mentioned that some women were concerned about the poor quality of services (e.g. shelters, training, food, etc.) provided by NGOs that offered them shelter. Returnees often had high expectations, which were difficult for NGOs to meet, leading to a high rate of moving between services. Returnees frequently moved between different shelters, seeking better training programs, food, or living conditions. This dissatisfaction with service quality ultimately contributed to challenges in long-term reintegration.

These women have such high expectations that they continuously ask for different training programs. They visit various organizations to compare services and often gossip about their experiences. One of the women mentioned that she had visited many organizations but found the services insufficient. (Participant of advocacy role)

The NGO workers explained that the lack of satisfaction was due to limited services, a constrained budget, and insufficient human resources. The low program budget was associated with understaffing, provision of a limited range of services for a limited number of women, limited community-based programs for returnees, families, and community members, and lack of programs to reach out to needy women and communities.

#### Lack of trust in NGO services

Some survivors’ families rejected both returnees and NGOs offering help, often believing that NGOs were motivated primarily by financial gain rather than genuine concern for survivors’ well-being. This mistrust also extended to the returnees themselves, who sometimes felt that NGOs were not fully committed to their recovery. One participant shared that one returnee was physically assaulted by her father and forbidden from staying in the shelter home.

It’s frustrating that families do not recognize the efforts NGOs make for the wellbeing of survivors and their reintegration into society. The survivor’s family often prevents them from staying in shelters, believing that NGOs are only interested in financial gain from donations. They fail to appreciate the valuable support that rehabilitation services provide. (Participant of program management role)

I learned that before she contacted me, her father had beaten her and did not want her to receive rehabilitation services or training. She was very distressed and said she could not stay with her father. I offered her a place in our shelter, and given the circumstances, she felt it was better to stay with us and agreed. (Participant of advocacy role)

#### Restricted living conditions within the NGO shelters

NGO shelters were described as having rigid, institutionalized environments, which were difficult for many returnees to adjust to. Participants noted that not all returnees felt a sense of belonging in NGO shelters, as life was quite different and highly institutional. Some returnee women were accustomed to very different lifestyles, such as those in big cities, or preferred more freedom in their dress and daily routines. The structured environment of the shelters, with its rules and limitations (e.g. time limits for going out), was challenging for some returnees to adapt to. Additionally, some returnees lacked interest in activities like career training and felt coerced into participating. Participants also feared that the coercive, institutional lifestyle in NGO shelters might be causing some women to have mixed feelings about returning to Nepal and staying in these shelters.

In Indian brothels, women may initially be coerced into sexual activities, but over time, they have freedom through their own earnings and the ability to buy things for themselves. Many of these women come to NGO shelters because they cannot stay in India. However, their freedom is restricted in the shelters. The structured routine and training at the shelters make it difficult for them to adapt to this institutional lifestyle, which is very different from what they are accustomed to. (Participant of program management role)

## Opportunities for the reintegration of sex-trafficking survivors into communities

Opportunities included factors or conditions that either facilitated the reintegration process for survivors or supported NGOs in successfully delivering reintegration programs, leading to more effective reintegration.

### Family support

Many participants reported successfully reintegrating several women and identified acceptance from the family as a crucial condition for successful reintegration. They described how the returnees’ families were generally contacted and invited to participate in parallel counseling sessions. In these sessions, both the returnees and their families were reintroduced to each other, supported in their reintegration needs, and provided counseling for any additional issues related to reintegration.

There are many cases where returnees are welcomed by their families, especially after the counseling sessions. Some families are easier to counsel, and we see their efforts to accept the survivors. I remember one person who was extremely supportive of his daughter. He was very happy to have her back. (Participant of program management role)

Participants noted that while some families were more cooperative and willing to accept the women upon their return, others were reluctant. In some cases, returnees were rejected by their families even after participating in counseling programs. Women who were abandoned by their families ended up staying in NGO shelters and continued with NGO-based employability programs.

### Returnee women working as social workers or activists

Participants believed that vocational and skill development training significantly impacted the lives of some women by providing opportunities to work in the anti-trafficking sector as social workers, activists, program officers, and secretaries. Participants with a history of sex trafficking often showed gratitude and a strong willingness to work with and for other survivors. Having survivors work as NGO staff created a supportive environment and fostered a better understanding among other women in the shelters. These women were seen as role models, inspiring others to pursue career and skill development opportunities.

We have created a supportive environment for returnees to stay in the shelters and to establish themselves as entrepreneurs. Some have worked with us in our programs, some have volunteered, and some have found permanent or temporary work with other anti-trafficking organizations. (Participant of program management role)

Coordination among NGOs provided opportunities for skill development and employment, especially for those not accepted by their families. As a result, returnee women, depending on their educational backgrounds, were able to volunteer in anti-trafficking activities, remained optimistic about job opportunities in anti-trafficking organizations, or received seed money to start their own businesses (e.g. retail shops, handmade crafts, beauty, personal care businesses, etc.) for livelihood.

### Coordinated support systems

Most participants highlighted the importance of receiving support from various institutions, particularly police, legal bodies, and healthcare providers, as critical for successful reintegration. They noted that there was existing coordination between some NGOs and health centers/hospitals to offer health services to returnee women. These services included general health screenings, safe abortion and delivery services for pregnant returnees, free medical supplies, emergency health support, and first aid services. Participants reported that health issues among returnees frequently involved sexually transmitted diseases, HIV, physical disabilities, and mental health problems such as trauma, post-traumatic stress disorder, depression, anxiety, and psychological distress.

Participants also mentioned that NGOs collaborated with legal organizations to provide consultations and advice, assist with filing cases, and help survivors obtain their rights and monetary compensation. They emphasized the need for cooperation with police and administrative bodies to support paperwork related to the registration of births, marriages, or divorces.

## Discussion

To our knowledge, this is the first study to explore the challenges and opportunities for the reintegration of survivors of sex trafficking into Nepali communities. The existing social and contextual barriers identified include a familial non-acceptance, pervasive social stigma, ineffective legal and support systems, and safety risks for both survivors and support providers. Within reintegration programs, factors such as poor service satisfaction, lack of trust, restrictive living conditions in shelters, and inadequate confidentiality practices hindered the program’s effectiveness. Opportunities for successful reintegration were found in familial support, employment as social workers or activists, and well-coordinated support systems, though these benefits were only accessible to a limited number of survivors.

Our study revealed that only a small portion of returnee women sought help from NGOs, while most either returned to live with their families, often facing stigma and rejection, or lived independently without engaging with NGOs or their families. A significant number of women requiring reintegration services remain unreached by current programs, and even those who enter the reintegration process encounter numerous challenges, making reintegration a multifaceted and prolonged journey. Broader social-contextual challenges, such as stigma and labeling, combined with program-related issues like service dissatisfaction, low trust, and confidentiality breaches, complicate reintegration efforts. Women who remain outside these programs, or drop out, often face physical and mental health issues, economic hardships, and the risk of being forced back into prostitution or survival sex, with a heightened risk of HIV infection and re-trafficking [16,20,21].

While some survivors reported positive experiences with reintegration programs, particularly through opportunities to work as social workers, addressing the challenges in program implementation is crucial for improving outcomes. Challenges such as denial of services and the social stigma attached to trafficking and prostitution must be addressed, as stigma not only hinders program workers but also directly affects survivors’ and their families’ participation in reintegration programs. In highly stigmatized social contexts, survivors often conceal their identity from program workers, friends, and family to avoid being labeled as ‘prostitutes’ or blamed for their exploitation [22]. Furthermore, institutionalized stigma and political protection for traffickers continue to impede effective assistance for survivors [11].

Considering these broader social-contextual factors, multi-sectoral reintegration interventions, including law enforcement activities and public health services such as primary care, sexual and reproductive health, and mental health support, are critical for successful reintegration [23]. Addressing institutional challenges, such as poor service satisfaction, lack of trust, restrictive living conditions, limited resources, and the stigma associated with NGO shelters, is essential. A shift from institution-based rehabilitation to community-based rehabilitation (i.e. de-institutionalization) could potentially improve satisfaction among survivors and increase participation while also reducing stigma [24,25]. The negative aspects of institutional rehabilitation must be carefully considered to avoid unintentionally violating the rights and dignity of returnees.

Family and social support are integral to achieving successful reintegration, yet preparing families to accept trafficking survivors is a complex task [22,26]. Participants in our study emphasized that counseling programs for both survivors and families could be an important first step in this process. Misinformation and lack of communication between service providers, survivors, and families can lead to mistrust and hinder reintegration efforts [22]. The institutionalization of survivors is often met with resistance due to stigma and labeling, underscoring the need for culturally sensitive, tailored counseling sessions that address anti-stigma issues [22,26]. Involving local leaders, women’s groups, health volunteers, and family members in the reintegration process, alongside political commitment and government support, is essential to secure adequate funding and ensure a coordinated, long-term approach to reintegration [27,28].

The role of NGO workers in combating stigma and their ability to deliver program content effectively are vital for overall program success. Mobilizing survivors to work with families and provide psychosocial and familial counseling can be an effective strategy for social reintegration. Economic reintegration, alongside psychosocial and social reintegration, is essential for ensuring the sustainability of reintegration efforts [29]. While many NGO-based programs offer income-generation support, these programs often have strict recruitment criteria, limiting access for many survivors. Coordinated efforts across various organizations to increase access to healthcare, build self-efficacy, and improve employability or entrepreneurial skills at the institutional, family, and community levels are essential for achieving successful outcomes in reintegration [23,29].

### Study limitations

The study has several limitations. First, the concentration of NGOs in Kathmandu and Lalitpur rather than the affected districts influenced site selection, as most reintegration programs are headquartered there. Our purposive sample from these two districts and one affected district limited our ability to include a broader geographical scope and achieve data saturation. Second, the study did not include participants from diverse educational levels and cultural backgrounds, potentially affecting the richness of the data. Consequently, the findings may not fully represent the broader population of survivors and their family members or capture all contextual nuances. Third, the findings could have been influenced by researchers’ subjectivity. However, the first author, who conducted and moderated all the interviews, is well-trained in qualitative research and aimed to maintain neutrality and minimize personal bias. We also sought to mitigate this limitation by including responses from reintegration workers with diverse backgrounds, which helped triangulate information from various perspectives.

Despite these limitations, the exploratory nature of the study and the qualitative method employed provide valuable insights and a foundation for future research and interventions in this area. Future research should address the absence of direct survivor perspectives in the present study through ethical, trauma-informed methodologies to capture their lived experiences and agency in the reintegration process.

## Conclusion

The reintegration of trafficking survivors is a complex, multifaceted process influenced by social, contextual, and programmatic factors. Key challenges to reintegration include a lack of familial acceptance, pervasive social stigma, ineffective legal and support systems, and safety risks for both survivors and service providers. Additionally, issues such as poor service satisfaction, lack of trust, restrictive living conditions in shelters, and inadequate confidentiality practices further impede the effectiveness of reintegration programs. Family support, employment opportunities, and well-coordinated support systems are critical enablers of successful reintegration. To improve outcomes, it is essential to secure adequate funding to scale up programs, address community stigma, and strengthen institutional capacities, ensuring more survivors are able to achieve successful reintegration.

## Data Availability

None (We do not share data due to the security of the participants).
